# Adverse childhood experiences and arthritis risk: exploring behavioral and psychological mediators in the behavioral risk factor surveillance system

**DOI:** 10.3389/fpubh.2025.1697021

**Published:** 2026-01-08

**Authors:** Qinxin Zhou, Jianzeng Shen, Dongdong Cao, Weijie Yu, Jixin Chen

**Affiliations:** 1 Department of Orthopaedic Surgery, Shaoxing Hospital of Traditional Chinese Medicine Affiliated to Zhejiang Chinese Medical University, Shaoxing, China; 2 Department of Orthopaedic Surgery, First Teaching Hospital of Tianjin University of Traditional Chinese Medicine, Tianjin, China; 3 Department of Orthopaedic Surgery, YunNan Provincial Hospital of Traditional Chinese Medicine, Kunming, China; 4 Department of Orthopaedic Surgery, Shaoxing Shangyu District Hospital of Traditional Chinese Medicine, Shaoxing, China

**Keywords:** adverse childhood experiences, arthritis, BRFSS, depression, mediation analysis

## Abstract

**Objective:**

This study examined the association between adverse childhood experiences (ACEs) and arthritis among U.S. adults and explores underlying biological and psychosocial mechanisms using data from the Behavioral Risk Factor Surveillance System (BRFSS).

**Methods:**

Data from the 2023 cycle of the BRFSS, managed by the CDC, was utilized to analyze the association between ACEs and arthritis. ACEs were categorized into five groups (0, 1, 2, 3, ≥4). Logistic regression models, adjusted for demographics, socio-economic status, and health behaviors, examined this association. Counterfactual-based mediation analyses estimated the extent to which smoking and depression mediate the ACE–arthritis relationship using logistic regression with 5,000 bootstrap samples.

**Results:**

The research included analysis of 32,594 adults, revealing significant differences in arthritis prevalence among individuals with varying categories of ACEs exposure. Higher ACE exposure was associated with increased odds of arthritis in a dose-response manner. In fully adjusted models, participants with ≥4 ACEs had 55% higher odds of arthritis (OR = 1.55, 95% CI: 1.43–1.67) compared with those with no ACEs. Smoking and depression partially mediated this association, accounting for 8.70 and 25.00% of the total effect, respectively.

**Conclusion:**

ACEs were associated with higher odds of arthritis, and this association was partially mediated by smoking and depression. The findings underscore the importance of addressing early-life adversity in public health strategies to reduce the long-term risk of arthritis.

## 1 Introduction

Arthritis, as one of the most common chronic diseases worldwide, significantly impacts the quality of life for a large number of adults (1). Both rheumatoid arthritis (RA), an autoimmune disease triggered by immune system abnormalities, and osteoarthritis (OA), a degenerative joint disease associated with aging and joint wear, impose substantial socioeconomic burdens (2). Although the pathogenesis of arthritis is influenced by a variety of factors, including genetics, environmental exposures, and immune dysregulation, the exact mechanisms and risk factors remain incompletely understood. In recent years, researchers have increasingly recognized the potential role of Adverse Childhood Experiences (ACEs) in shaping long-term health, particularly their strong association with the development of various chronic diseases.

ACEs refer to traumatic events experienced during childhood, such as domestic violence, emotional neglect, parental divorce, the loss of a loved one, or other forms of adversity (3). A growing body of evidence suggests that ACEs have lasting effects on an individual's psychological wellbeing, social adaptation, and physical health (4). Through interactions between the immune, endocrine, and nervous systems, ACEs may elevate the risk of numerous chronic conditions, including cardiovascular diseases, depression, and diabetes (5). Beyond biological pathways linking ACEs to chronic disease, sex may shape both health outcomes and the disclosure of ACEs. Prior work suggests sex differences in arthritis burden and depressive symptoms, and men and women may differ in reporting or recognizing childhood adversity (6, 7). These differences can influence exposure ascertainment and observed associations. However, research investigating the specific association between ACEs and arthritis remains limited, with existing studies often restricted to small sample sizes or specific populations (8).

To address this research gap, the present study utilizes data from the Behavioral Risk Factor Surveillance System (BRFSS), the largest behavioral health survey database in the United States. The BRFSS database encompasses a diverse adult population and provides comprehensive data on behaviors, health conditions, and diseases, offering high representativeness and broad applicability. By analyzing variables related to ACEs and arthritis within the BRFSS database, this study aims to explore the potential impact of ACEs on arthritis risk and to investigate underlying biological and psychosocial mechanisms (9). The findings are expected to provide theoretical insights for public health interventions and the early prevention of arthritis.

## 2 Methods

### 2.1 Data

This study leverages de-identified, publicly accessible data from the Behavioral Risk Factor Surveillance System (BRFSS), which is overseen by the Centers for Disease Control and Prevention (CDC) (10). The BRFSS systematically collects data on health-related behaviors, chronic conditions, and the utilization of healthcare services across states. Managed by state health departments, the survey data is forwarded to the CDC for analysis and data processing. Using a random-digit-dialing method, interviews are conducted via both landline and mobile phones. The survey participants are adults aged 18 or older who own a functioning telephone. Each state implements a core questionnaire, with certain items repeated every year and others every 2 years. States can also opt to include additional modules targeting specific health topics in their surveys. The data utilized in this analysis come from the 2023 BRFSS cycle. The Institutional Review Board at the principal investigators' institution has deemed the use of de-identified BRFSS data as not involving human subjects' research (11).

### 2.2 Measures

ACEs. There are a total of eleven questions originating from the CDC-Kaiser ACEs study. The eleven questions encompass eight domains of adverse experiences including emotional abuse, sexual abuse, physical abuse, parental separation/divorce, witnessing intimate partner violence, incarcerated household member, substance abuse in the household, and mental illness in the household. The number of “yes” responses was summed to calculate the total ACEs score, ranging from 0 to 11 (12). The ACEs score is the number of ACEs types experienced and is divided into five categories of 0,1,2,3, and ≥4 (13). By dividing the ACEs score into five categories, we sought to clearly differentiate the risk associated with the cumulative number of ACEs experienced. The term “ACEs category 1” specifically refers to individuals who reported experiencing one ACEs. Similarly, “ACEs category 0” refers to individuals who reported experiencing 0 ACEs, and this consistent naming scheme is applied to categories 2, 3, and ≥4. The widely used 0/1/2/3/≥4 categorization was originally proposed by Felitti et al. (14) and has been adopted in the majority of subsequent BRFSS-based ACE studies for several reasons: (1) it allows for comparability across studies using BRFSS data; (2) it captures the pronounced dose-response relationship with chronic disease outcomes; (3) it identifies a clinically meaningful threshold at ≥4 ACEs, where previous research has consistently demonstrated markedly elevated risk for multiple adverse health outcomes; and (4) it balances statistical precision with adequate sample sizes in each category.

Arthritis diagnosis among participants is confirmed through their response to whether a doctor has diagnosed them with some form of arthritis.

Covariates. In this study, a wide range of covariates was used to explore the potential link between adverse childhood experiences and the risk of arthritis in adulthood. The covariates encompassed both biomedical and socio-economic indicators, including basic demographics such as gender (male, female), age groups (18–24, 25–34, 35–44, 45–54, 55–64, over 65), and race/ethnicity (White only, Non-Hispanic; Black only, Non-Hispanic; Other race only, Non-Hispanic; Multiracial, Non-Hispanic; Hispanic; Other). Socio-economic status was assessed through variables such as education level (below high school, high school, more than high school), annual income (< $25,000, $25,000 to $50,000, more than $50,000), and employment status (employed, unemployed, retired, unable to work). Health-related behaviors were also included, such as smoking status (every day, some days, former, never), binge drinking (yes, no), and physical activity (met both guidelines, met aerobic guidelines only, met strengthening guidelines only, did not meet either guideline). Smoking status was classified as: current smoker every day (currently smoking cigarettes every day), current smoker some days (currently smoking cigarettes on some days), former smoker (smoked at least 100 cigarettes in lifetime but not currently smoking), and never smoker (smoked fewer than 100 cigarettes in lifetime). Binge drinking is defined according to the CDC guidelines as consuming five or more alcoholic drinks on a single occasion for men, or 4 or more drinks for women, within the past 30 days. Physical Activity Guidelines for Americans recommends at least 150 min of moderate-intensity aerobic activity or 75 min of vigorous-intensity aerobic activity per week, along with muscle-strengthening activities on two or more days per week (15). Additional health conditions were accounted for by including BMI categories (underweight, normal weight, overweight, obese), diabetes status (yes, no), health insurance coverage (yes, no), urban/rural status (urban, rural), and the presence of depression (yes, no). Body Mass Index (BMI) was calculated from self-reported height and weight (kg/m^2^) and categorized according to WHO standards: underweight (< 18.5 kg/m^2^), normal weight (18.5–24.9 kg/m^2^), overweight (25.0–29.9 kg/m^2^), and obese (≥30.0 kg/m^2^). The presence of depression refers to a self-reported diagnosis of depressive disorder, which includes major depression, dysthymia, and minor depression. Diabetes refers to self-reported diagnoses of diabetes. Participants who were told by a healthcare professional that they had diabetes, either Type 1 or Type 2, were considered to have diabetes. Additionally, for female respondents, we considered gestational diabetes (diabetes diagnosed only during pregnancy) if it was explicitly mentioned by the respondent. Urban/rural status was determined using the BRFSS metropolitan statistical area (MSA) classification, with urban defined as residing within a Metropolitan Statistical Area and rural as residing outside an MSA. This comprehensive set of covariates helps control for potential confounders and provides insights into the complex relationships between adverse childhood experiences and later-life arthritis risk (16, 17).

### 2.3 Data synthesize and statistical analysis

Continuous data following a normal distribution were reported using means and standard deviations (SD). Categorical variables were expressed in frequencies (percentages). Categorical data analysis was conducted using Pearson's chi-square test, while continuous data were analyzed using one-way ANOVA. We investigated the association between ACEs and arthritis through logistic regression models, with adjustments for potential confounders and without. Interaction effects were assessed based on variables such as gender, age, educational level, diabetes status, smoking habits, and physical activity levels (18, 19). Mediation analyses were conducted using the counterfactual framework implemented in the R package “mediation.” We fitted logistic regression models for both the outcome (arthritis) and mediators (current smoking, lifetime depression diagnosis). Indirect effects were estimated with 5,000 bootstrap replications. All effects are reported on the log-odds scale. Proportion mediated was calculated as indirect effect/total effect (20). All statistical analyses were performed using R version 4.2.3, with statistical significance defined by a two-sided *P*-value < 0.05.

## 3 Results

### 3.1 Participant characteristics

**Figure 1:**
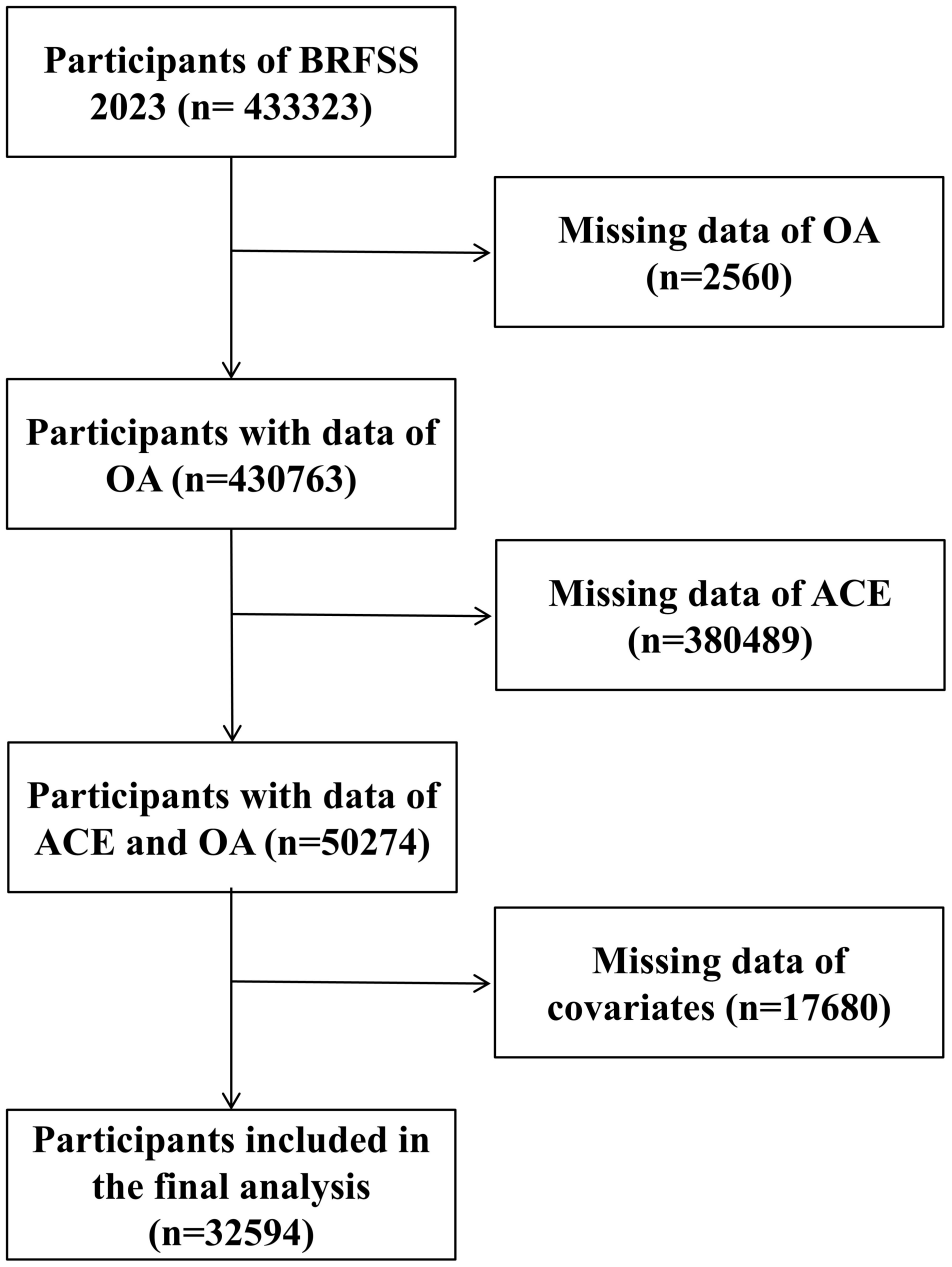
Flowchart of participants included in the analysis.

This research exploring the association between ACEs and arthritis among 32,594 adults revealed significant demographic, socioeconomic, and health-related differences between participants with and without arthritis, detailed in Figure 1 and Table 1. The arthritis group, older on average, had a higher proportion of females and Non-Hispanic white people, with these differences achieving statistical significance (*P* < 0.001). Socioeconomic analysis showed that individuals with arthritis generally reported lower income levels and education, and were less likely to be employed, with a notable number reporting being unable to work (*P* < 0.001). Health behaviors and conditions also varied significantly; those with arthritis had a higher mean BMI, were more likely to smoke, and had higher rates of diabetes and depression (*P* < 0.001). Furthermore, the mean ACEs score was modestly higher in the arthritis group, suggesting a potential linkage between ACEs and arthritis onset (*P* < 0.001).

**Table 1:** Demographic characteristics of participants.

**Characteristic**	**Overall**	**Arthritis**	**No arthritis**	***x*^2^/*t*/*z***	***P-*value**
*N*	32,594	11,678	20,916		
BMI	27.46 (24.33–31.75)	28.59 (25.06–33.20)	27.12 (23.92–31.01)	0.245	< 0.001
**Race**					
White only, non-hispanic	25,020 (76.76%)	9,532 (81.62%)	15,488 (74.05%)	0.248	< 0.001
Black only, non-hispanic	3,228 (9.90%)	1,116 (9.56%)	2,112 (10.10%)		
Other race only, non-hispanic	1,122 (3.44%)	269 (2.30%)	853 (4.08%)		
Multiracial, non-hispanic	691 (2.12%)	236 (2.02%)	455 (2.18%)		
Hispanic	2,206 (6.77%)	408 (3.49%)	1,798 (8.60%)		
Other	327 (1.00%)	117 (1.00%)	210 (1.00%)		
**Gender**					
Male	15,679 (48.10%)	4,817 (41.25%)	10,862 (51.93%)	0.215	< 0.001
Female	16,915 (51.90%)	6,861 (58.75%)	10,054 (48.07%)		
**Age**					
18–24	1,319 (4.05%)	57 (0.49%)	1,262 (6.03%)	0.844	< 0.001
25–34	3,120 (9.57%)	245 (2.10%)	2,875 (13.75%)		
35–44	3,995 (12.26%)	625 (5.35%)	3,370 (16.11%)		
45–54	4,597 (14.10%)	1,239 (10.61%)	3,358 (16.05%)		
55–64	6,212 (19.06%)	2,453 (21.01%)	3,759 (17.97%)		
Over 65	13,351 (40.96%)	7,059 (60.45%)	6,292 (30.08%)		
**Marital**					
Married	18,797 (57.67%)	6,439 (55.14%)	12,358 (59.08%)	0.080	< 0.001
Unmarried	13,797 (42.33%)	5,239 (44.86%)	8,558 (40.92%)		
**Education**					
Blow high school	1,455 (4.46%)	640 (5.48%)	815 (3.90%)	0.118	< 0.001
High school	7,467 (22.91%)	2,934 (25.12%)	4,533 (21.67%)		
More than high school	23,672 (72.63%)	8,104 (69.40%)	15,568 (74.43%)		
**Diabetes**					
Yes	5,258 (16.13%)	2,738 (23.45%)	2,520 (12.05%)	0.302	< 0.001
No	27,336 (83.87%)	8,940 (76.55%)	18,396 (87.95%)		
**Income**, ***n*** **(%)**					
Less than 25,000	4,260 (13.07%)	2,013 (17.24%)	2,247 (10.74%)	0.278	< 0.001
25,000 to 50,000	7,630 (23.41%)	3,230 (27.66%)	4,400 (21.04%)		
More than 50,000	20,704 (63.52%)	6,435 (55.10%)	14,269 (68.22%)		
**Employment**					
Employed	16,311 (50.04%)	3,608 (30.90%)	12,703 (60.73%)	0.662	< 0.001
Unemployment	1,088 (3.34%)	296 (2.53%)	792 (3.79%)		
Retired	11,698 (35.89%)	6,171 (52.84%)	5,527 (26.42%)		
Unable to work	3,497 (10.73%)	1,603 (13.73%)	1,894 (9.06%)		
**Smoker**					
Every day	2,714 (8.33%)	1,120 (9.59%)	1,594 (7.62%)	0.258	< 0.001
Some day	1,014 (3.11%)	372 (3.19%)	642 (3.07%)		
Former	9,743 (29.89%)	4,262 (36.50%)	5,481 (26.20%)		
Never	19,123 (58.67%)	5,924 (50.73%)	13,199 (63.10%)		
**BMI group**					
Underweight	472 (1.45%)	158 (1.35%)	314 (1.50%)	0.249	< 0.001
Normal weight	9,180 (28.16%)	2,692 (23.05%)	6,488 (31.02%)		
Overweight	11,846 (36.34%)	4,017 (34.40%)	7,829 (37.43%)		
Obese	11,096 (34.04%)	4,811 (41.20%)	6,285 (30.05%)		
**Health insurance**					
Yes	31,029 (95.20%)	11,417 (97.77%)	19,612 (93.77%)	0.200	< 0.001
No	1,565 (4.80%)	261 (2.23%)	1,304 (6.23%)		
**Urban/rural status**					
Urban status	29,322 (89.96%)	10,174 (87.12%)	19,148 (91.55%)	0.144	< 0.001
Rural status	3,272 (10.04%)	1,504 (12.88%)	1,768 (8.45%)		
**Binge drinking**					
No	28,338 (86.94%)	10,648 (91.18%)	17,690 (84.58%)	0.203	< 0.001
Yes	4,256 (13.06%)	1,030 (8.82%)	3,226 (15.42%)		
**Physical activity**					
Met both guidelines	10,028 (30.77%)	3,256 (27.88%)	6,772 (32.38%)	0.193	< 0.001
Met aerobic guidelines only	10,589 (32.49%)	3,497 (29.95%)	7,092 (33.91%)		
Met strengthening guidelines only	3,613 (11.08%)	1,310 (11.22%)	2,303 (11.01%)		
Did not meet either guideline	8,364 (25.66%)	3,615 (30.96%)	4,749 (22.71%)		
**ACEs category**					
0	10,949 (33.59%)	3,867 (33.11%)	7,082 (33.86%)	0.049	0.001
1	7,330 (22.49%)	2,522 (21.60%)	4,808 (22.99%)		
2	4,428 (13.59%)	1,595 (13.66%)	2,833 (13.54%)		
3	3,087 (9.47%)	1,144 (9.80%)	1,943 (9.29%)		
≥4	6,800 (20.86%)	2,550 (21.84%)	4,250 (20.32%)		
ACEs score	1.97 ± 2.29	2.04 ± 2.33	1.94 ± 2.26	0.044	< 0.001
**Depression**					
Yes	6,641 (20.37%)	3,158 (27.04%)	3,483 (16.65%)	0.253	< 0.001
No	25,953 (79.63%)	8,520 (72.96%)	17,433 (83.35%)		

### 3.2 Associations between ACEs and arthritis

Table 2 displays the associations between ACEs and the risk of arthritis. Across all three models, different ACEs categories are significantly associated with arthritis. In Model 3, which adjusts for all confounders, the odds ratios for arthritis increase with the ACEs categories from 0 to 4 as follows: 1.04 (95% CI: 0.97–1.12), 1.21 (95% CI: 1.12–1.31), 1.34 (95% CI: 1.22–1.48), and 1.55 (95% CI: 1.43–1.67). However, the association between ACEs category 1 and arthritis is not significant after further adjustment for all variables including other confounders (OR = 1.04, 95% CI: 0.97–1.12, *P* = 0.229). When considering the ACEs score as a continuous variable, the odds of developing arthritis increase by 1.02 times (95% CI: 1.01–1.03, *P* < 0.001) in Model 1, 1.13 times (95% CI: 1.12–1.14, *P* < 0.001) in Model 2, and 1.08 times (95% CI: 1.07–1.10, *P* < 0.001) in Model 3 for each unit increase in the ACEs score.

**Table 2:** Association of ACEs category and ACEs score with arthritis.

**Exposure**	**Model 1**		**Model 2**		**Model 3**	
**ACEs category**	**OR (95%CI)**	***P*** **-value**	**OR (95%CI)**	***P*** **-value**	**OR (95%CI)**	***P*** **-value**
0	1.0		1.0		1.0	
1	0.96 (0.90, 1.02)	0.203	1.10 (1.03, 1.18)	0.005	1.04 (0.97, 1.12)	0.229
2	1.03 (0.96, 1.10)	0.464	1.33 (1.23, 1.44)	< 0.001	1.21 (1.12, 1.31)	< 0.001
3	1.09 (1.01, 1.19)	0.032	1.56 (1.42, 1.71)	< 0.001	1.34 (1.22, 1.48)	< 0.001
≥4	1.10 (1.04, 1.17)	0.002	1.99 (1.85, 2.14)	< 0.001	1.55 (1.43, 1.67)	< 0.001
ACEs score	1.02 (1.01, 1.03)	< 0.001	1.13 (1.12, 1.14)	< 0.001	1.08 (1.07, 1.10)	< 0.001

Model 1: crude mode; Model 2: adjusted for age, gender, education, race, marital status, income; Model 3: adjusted for age, gender, education, race, marital status, income, employment, BMI, drink, smoke, diabetes, health insurance, urban and rural status, physical activity, depression.

### 3.3 Subgroup analysis

**Figure 2:**
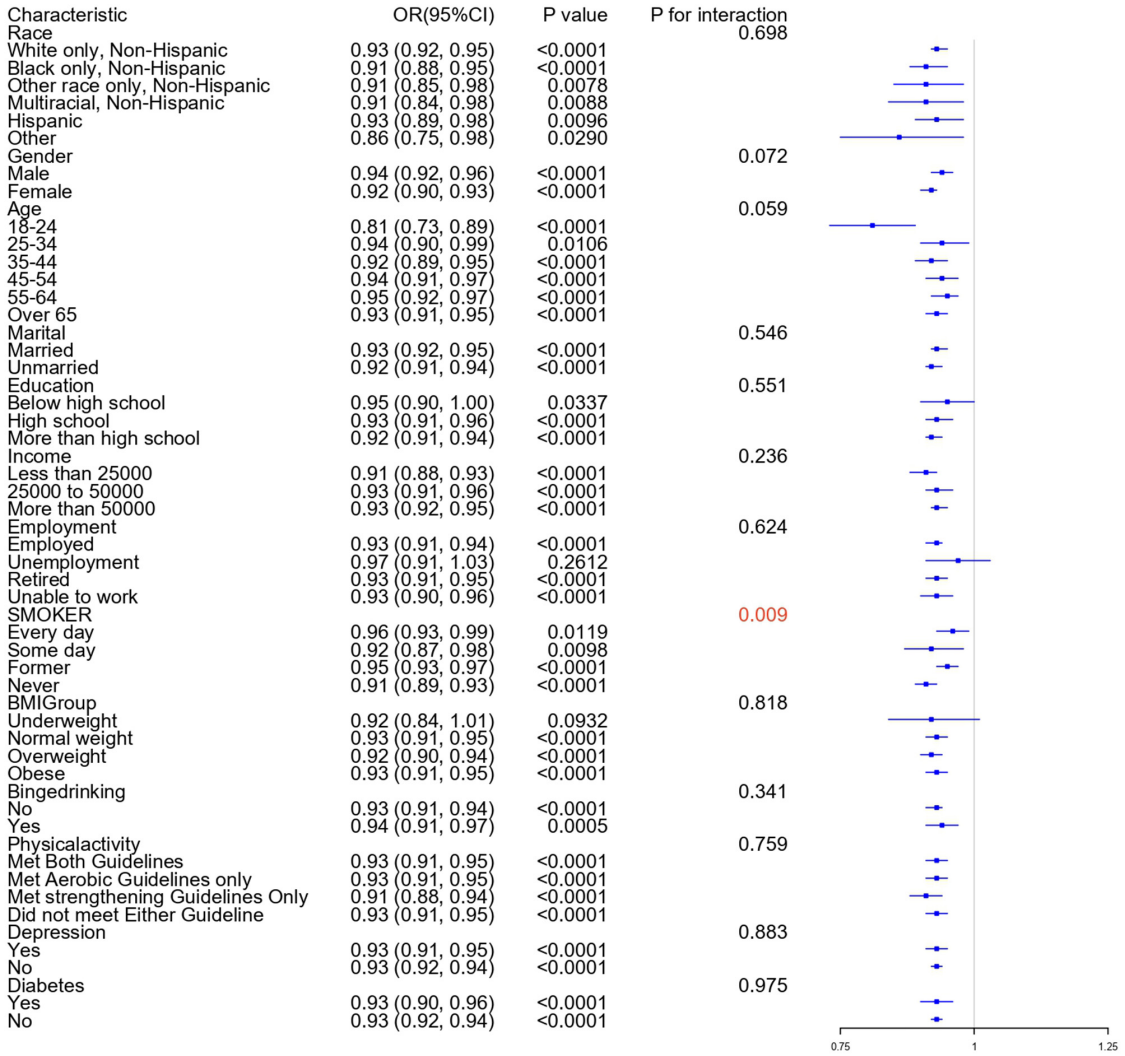
Subgroups analysis of ACEs score and arthritis. Forest plot showing odds ratios (ORs) and 95% confidence intervals for the association between ACEs score and arthritis, stratified by demographic and health-related subgroups. Each OR represents the change in arthritis odds for each additional ACE reported. All models were adjusted for demographics, socioeconomic status, and health behaviors, except for the stratification variable. *P* for interaction was calculated using likelihood ratio tests comparing models with and without interaction terms. The significant interaction with smoking (*P* = 0.009, shown in red) indicates that smoking status modifies the strength of the ACEs-arthritis association.

Subgroup analyses examined whether the association between ACEs score and arthritis varied across demographic and health-related strata. The analyses were stratified by age, gender, education level, race, marital status, income, employment status, BMI, drinking, smoking, diabetes, health insurance, urban/rural status, physical activity, and depression symptoms. As shown in Figure 2, the association between ACEs and arthritis was consistently positive across all subgroups, with ORs ranging from 0.86 to 0.97 per unit increase in ACEs score. Most stratifications did not significantly modify this association (all *P* for interaction >0.05), indicating the relationship is robust across different population segments. However, a significant interaction was observed with smoking behavior (*P* = 0.009), suggesting that the magnitude of the ACEs-arthritis association differs by smoking status, with non-smokers (OR = 0.91) showing a slightly stronger association compared to current daily smokers (OR = 0.96).

### 3.4 Mediation analysis

**Figure 3:**
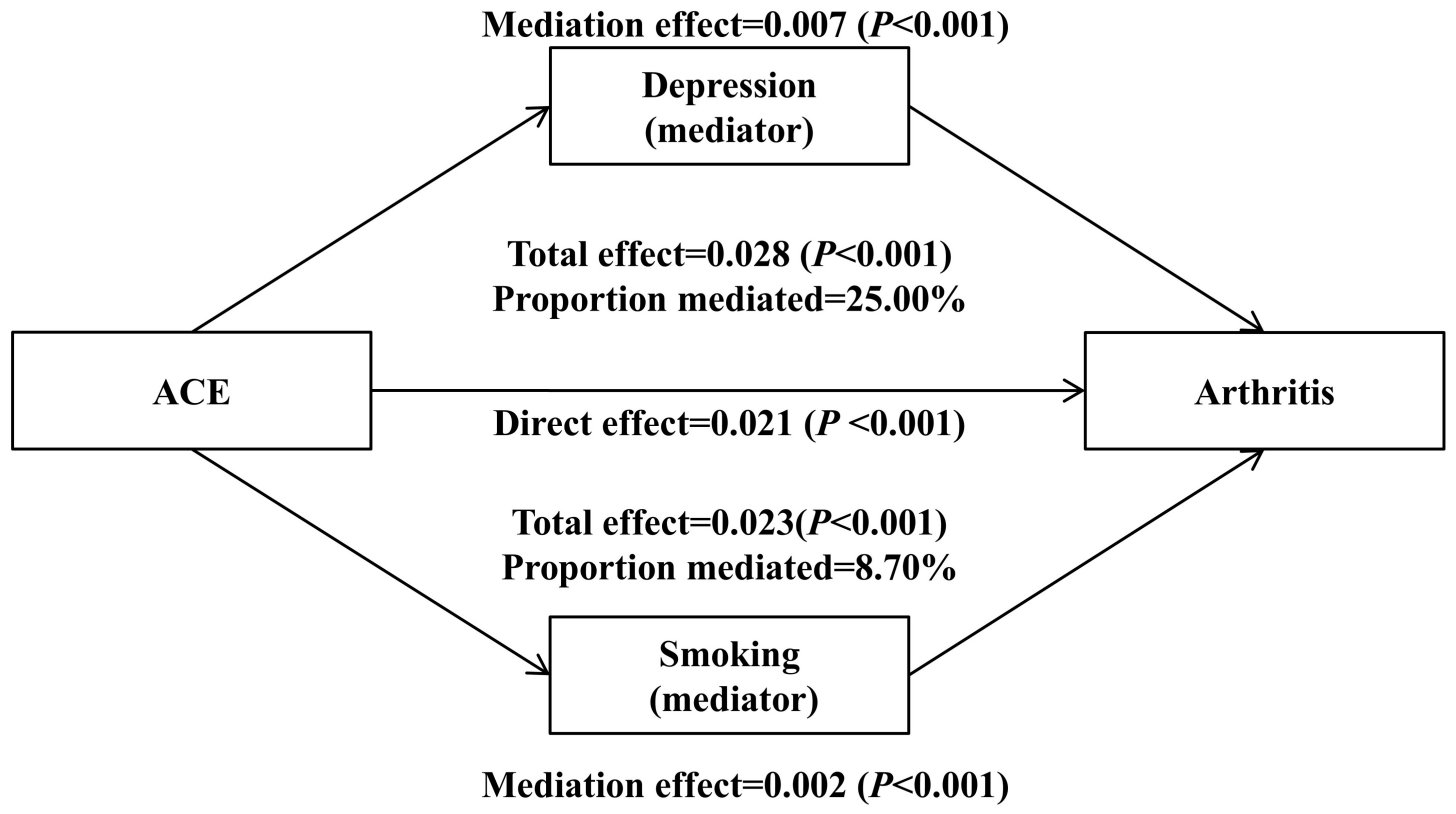
Mediation analysis showing the pathways through which smoking and depression mediate the relationship between ACEs (exposure) and arthritis (outcome). The diagram illustrates: (1) Direct effects—the association between ACEs and arthritis not mediated through smoking or depression (path c'); (2) Indirect effects – the association between ACEs and arthritis that operates through smoking (path a1 × b1) and depression (path a_2_ × b_2_); and (3) Proportion mediated—the percentage of the total ACEs-arthritis association explained by each mediator. All effect estimates are reported on the log-odds scale with 95% confidence intervals derived from 5,000 bias-corrected bootstrap replications. Models were adjusted for age, gender, education, race, marital status, income, employment, BMI, drinking status, diabetes, health insurance, urban/rural status, and physical activity. Results show that depression (25.00% mediation) has a stronger mediating effect than smoking (8.70% mediation).

The results revealed that smoking partially mediated the association between ACEs and arthritis, with an indirect effect estimate of 0.002 (95% CI: 0.001–0.005, *P* < 0.001), accounting for 8.70% of the total effect (total effect: 0.023, 95% CI: 0.020–0.027, *P* < 0.001). The direct effect of ACEs on arthritis through pathways other than smoking remained significant (estimate: 0.021, 95% CI: 0.018–0.024, *P* < 0.001). Similarly, depression was identified as a significant mediator in the relationship between ACEs and arthritis. The indirect effect through depression was 0.007 (95% CI: 0.006–0.010, *P* < 0.001), which accounted for 25.00% of the total effect (total effect: 0.028, 95% CI: 0.025–0.031, *P* < 0.001). The direct effect of ACEs on arthritis, independent of depression, also remained significant (estimate: 0.021, 95% CI: 0.018–0.024, *P* < 0.001). These findings suggest that both smoking and depression partially mediate the relationship between ACEs and arthritis, with depression exhibiting a stronger mediating effect compared to smoking. For specific results and visual representation, refer to Table 3 and Figure 3.

**Table 3:** Effect of the smoking and depression (mediators) on the relationship between ACEs (exposure) and arthritis (outcome).

**Cohort**	**Direct effect**		**Indirect effect**		**Total effect**		**Proportion**	
	**Estimate (95% CI)**	***P*** **-value**	**Estimate (95% CI)**	***P*** **-value**	**Estimate (95% CI)**	***P*** **-value**	**Mediated**	***P*** **-value**
Smoking	0.021 (0.018, 0.024)	< 0.001	0.002 (0.001, 0.005)	< 0.001	0.023 (0.020, 0.027)	< 0.001	8.70%	< 0.001
Depression	0.021 (0.018, 0.024)	< 0.001	0.007 (0.006, 0.010)	< 0.001	0.028 (0.025, 0.031)	< 0.001	25.00%	< 0.001

All effect estimates are reported on the log-odds scale. Models were adjusted for age, gender, education, race, marital status, income, employment, BMI, drinking status, diabetes, health insurance, urban/rural status, and physical activity. Confidence intervals were estimated using bias-corrected bootstrap method with 5,000 replications. Proportion mediated = indirect effect/total effect.

## 4 Discussion

This study investigates the relationship between ACEs and the risk of arthritis using a large, representative sample of 32,594 adults from the BRFSS database (21). The findings reveal a significant positive association between ACEs and arthritis, with a clear dose-response relationship: higher ACE categories are linked to an increased risk of developing arthritis, even after adjusting for demographic, socioeconomic, and behavioral confounders. Additionally, this study provides novel evidence that smoking and depression partially mediate this relationship, with depression exerting a stronger mediating effect. These results contribute to a deeper understanding of how early-life adversity influences long-term health outcomes by uncovering pathways through which ACEs increase arthritis risk, including chronic inflammation, behavioral factors, psychosocial stress, and neuroimmune interactions. These findings emphasize the critical need for public health strategies addressing early-life adversity, mental health, and modifiable behaviors to reduce the burden of arthritis and improve long-term health outcomes.

The analysis demonstrates a dose-response relationship between ACEs and arthritis risk (13, 18). As ACEs categories increase, the odds of developing arthritis rise significantly, even after adjusting for demographic, socioeconomic, and behavioral confounders. This suggests that cumulative exposure to childhood adversity may contribute to the biological and psychosocial pathways that predispose individuals to arthritis. Notably, while ACEs category 1 was not significantly associated with arthritis after full adjustment, higher ACEs categories (2–4) showed a clear and significant association, reinforcing the idea that cumulative adversity is associated with increased arthritis risk. However, this study does not allow for conclusions about whether specific types of ACEs have differing impacts on arthritis. Additionally, the subgroup analysis revealed that the association between ACEs and arthritis remains consistent across most demographic and health-related strata, with the exception of smoking behavior (22). The significant interaction with smoking (*P* = 0.009) indicates that smoking status modifies the strength of the ACEs-arthritis relationship. Specifically, the association appears slightly attenuated among current smokers compared to never-smokers, which may reflect competing biological pathways or ceiling effects where smoking's direct pro-inflammatory effects partially mask or overshadow the ACEs-mediated inflammatory processes (19, 23). This finding underscores the complex interplay between early-life stress and adult health behaviors, and highlights the importance of considering behavioral modifiers when examining long-term health consequences of childhood adversity. These results suggest that smoking cessation interventions may be particularly beneficial for individuals with high ACEs exposure.

The observed association between ACEs and arthritis can be explained through multiple interrelated biological and psychosocial mechanisms, which may act independently or synergistically. Early-life stress disrupts the hypothalamic-pituitary-adrenal (HPA) axis and autonomic nervous system, resulting in heightened cortisol levels and immune dysregulation (24, 25). This leads to a pro-inflammatory state characterized by elevated cytokines such as tumor necrosis factor-alpha (TNF-α) and interleukin-6 (IL-6), which are directly implicated in the pathogenesis of inflammatory arthritis. Prolonged immune activation can cause tissue damage, joint degeneration, and increased susceptibility to autoimmune diseases like rheumatoid arthritis. ACEs may lead to epigenetic modifications, such as DNA methylation, that alter gene expression related to inflammation, stress response, and immune regulation (26). These modifications can have long-lasting effects, predisposing individuals to chronic inflammatory conditions like arthritis. Repeated exposure to stress during childhood increases allostatic load, the cumulative physiological toll of chronic stress. This burden impairs the body's adaptive capacity and promotes systemic inflammation, which accelerates wear and tear on tissues, including cartilage and joint structures, increasing arthritis risk. ACEs are linked to high-risk behaviors, including smoking, physical inactivity, and poor dietary habits, all of which promote systemic inflammation or joint damage (23). Smoking, for instance, increases oxidative stress and cartilage degradation, partially explaining its mediating role in the ACE-arthritis relationship. Studies show that smoking promotes oxidative stress and proteoglycan degradation in cartilage through pathways such as the aryl hydrocarbon receptor activation (27), and generates malondialdehyde as an oxidative stress marker correlating with cartilage damage (28). Advanced protein oxidation due to smoking is also linked with increased pain perception and cartilage degradation (29). ACEs significantly increase the risk of depression and mental health disorders, which exacerbate arthritis through heightened pain perception, systemic inflammation, and reduced self-care. Depression has been identified as a central mediator translating early-life adversity into arthritis onset (30). Emerging evidence suggests ACEs sensitize neural pathways associated with pain, increasing pain perception and the likelihood of chronic pain conditions, including arthritis (31). Altered neuroimmune interactions may also perpetuate inflammation, contributing to arthritis onset and progression.

This study's findings are largely consistent with prior research that has demonstrated a significant link between ACEs and chronic diseases, including arthritis. Researchers previously established that ACEs increase the risk of chronic inflammatory and autoimmune conditions, emphasizing the role of systemic inflammation and psychosocial stress as key mediators (12, 21). However, our findings diverge from some studies, which suggested that the association between ACEs and arthritis weakens after controlling for behavioral factors like smoking and physical activity. This discrepancy may be attributed to differences in statistical modeling or the inclusion of mediators like depression in our study, which strengthens the understanding of independent effects. Our findings provide a nuanced perspective by highlighting the stronger mediating role of depression compared to smoking, suggesting that psychological pathways are crucial in linking ACEs to arthritis.

The mediation analysis further elucidates the pathways through which ACEs influence arthritis development. Smoking and depression were identified as partial mediators, with depression exhibiting a stronger mediating effect than smoking (32). Depression accounted for nearly one-quarter of the total effect of ACEs on arthritis, underscoring the critical role of mental health in translating early-life adversity into physical disease (33). Smoking, as a modifiable risk factor, mediated a smaller yet significant proportion of the association (34). These findings highlight the need for targeted interventions to address both smoking and depression among individuals with high ACEs exposure to mitigate their risk of arthritis.

These findings underscore the importance of adopting a life-course approach to arthritis prevention and management. Interventions aimed at reducing ACEs exposure or mitigating its long-term effects could play a pivotal role in curbing the burden of arthritis. Screening for ACEs in clinical settings, particularly among individuals presenting with mental health conditions or high-risk behaviors like smoking, could facilitate early identification of at-risk populations. Additionally, integrating mental health support and smoking cessation programs into arthritis care could yield significant benefits. Future research should further investigate the biological mechanisms linking ACEs to arthritis, focusing on inflammatory pathways, epigenetic changes, and neuroimmune interactions. Longitudinal studies are needed to establish causal relationships and examine the efficacy of interventions targeting mediating factors. Exploration of additional mediators, such as physical activity and diet, would also enrich our understanding of this complex relationship.

Although this study has several strengths, including a large nationally representative sample, comprehensive covariate adjustment, and novel mediation analyses, important limitations must be acknowledged. First, the cross-sectional design precludes establishment of temporality or causality; we cannot definitively determine whether ACEs preceded arthritis onset, and reverse causation remains possible (e.g., individuals with chronic pain or disability from arthritis may be more likely to recall or report negative childhood experiences, or arthritis-related depression may bias ACE recall). Longitudinal studies with prospective assessment of both ACEs and arthritis incidence are needed to establish temporal relationships more definitively. Second, both ACEs and arthritis diagnoses rely on self-report and are therefore subject to recall bias and social desirability bias, particularly for sensitive childhood experiences. ACEs may be underreported due to memory decay, shame, or reluctance to disclose traumatic experiences, which would likely bias our estimates toward the null and suggest that the true associations may be even stronger than observed. Similarly, self-reported arthritis diagnosis may not distinguish between different arthritis subtypes or severity levels. Third, we could not distinguish arthritis subtypes (rheumatoid arthritis vs. osteoarthritis vs. others), which may have distinct etiologies and different relationships with ACEs. Future research should examine whether ACEs differentially impact inflammatory vs. degenerative arthritis. Fourth, despite extensive covariate adjustment, residual confounding by unmeasured factors remains possible. Variables such as genetic predisposition to both mental health conditions and arthritis, detailed early-life socioeconomic position (beyond adult SES), childhood physical illness or disability, access to childhood healthcare, and other unmeasured behavioral or environmental factors may partially explain the observed associations. Fifth, the BRFSS response rate, while typical for telephone-based surveillance surveys, raises the possibility of non-response bias. Individuals who experienced severe childhood adversity or have significant health problems may be less likely to participate in telephone surveys, potentially leading to underestimation of the true associations. Finally, our mediation analysis, while informative, has important limitations. The mediators (smoking and depression) were measured concurrently with the outcome, precluding definitive causal inference about mediation pathways. Additionally, we examined only two potential mediators; other unmeasured factors (e.g., chronic inflammation biomarkers, health service utilization, other health behaviors) may also mediate the ACEs-arthritis relationship.

Despite these limitations, our findings contribute important insights into the long-term health consequences of childhood adversity and highlight modifiable pathways (smoking cessation, depression treatment) that may reduce arthritis risk among individuals with high ACEs exposure. Future research should employ prospective designs, biomarker assessments, and more comprehensive mediation analyses to further elucidate these relationships.

## 5 Conclusion

This study highlights a significant association between ACEs and arthritis, with both smoking and depression identified as partial mediators. Furthermore, the mechanisms linking ACEs to arthritis underscore the interplay between chronic inflammation, immune dysregulation, epigenetic changes, and psychosocial factors. These findings emphasize the need for comprehensive public health strategies addressing early-life adversity, mental health, and modifiable risk behaviors to reduce the prevalence and impact of arthritis. By addressing the root causes of this relationship, healthcare providers and policymakers can better support individuals affected by ACEs and improve long-term health outcomes.

## Data Availability

The raw data supporting the conclusions of this article will be made available by the authors, without undue reservation.

## References

[1] Joshi S Reid MC Mindlis I. Illness intrusiveness, perceived control, and quality of life in older adults with arthritis and multimorbidity. Clin Gerontol. (2025) 48:1308–1319. doi: 10.1093/geroni/igae098.4215 39840595 PMC12280086

[2] Arden NK Perry TA Bannuru RR Bruyère O Cooper C Haugen IK . Non-surgical management of knee osteoarthritis: comparison of ESCEO and OARSI 2019 guidelines. Nat Rev Rheumatol. (2021) 17:59–66. doi: 10.1038/s41584-020-00523-9 33116279

[3] Von Korff M Alonso J Ormel J Angermeyer M Bruffaerts R Fleiz C . Childhood psychosocial stressors and adult onset arthritis: broad spectrum risk factors and allostatic load. Pain. (2009) 143:76–83. doi: 10.1016/j.pain.2009.01.034 19251363 PMC2703588

[4] Neufeld KM Karunanayake CP Maenz LY Rosenberg AM. Stressful life events antedating chronic childhood arthritis. J Rheumatol. (2013) 40:1756–65. doi: 10.3899/jrheum.121505 23950190

[5] Luiz AP Antico H Santos TA Nisihara R Skare TL. AB1351 systemic rheumatic diseases and cumulative childhood stress. Ann Rheum Dis. (2018) 77:1764. doi: 10.1136/annrheumdis-2018-eular.4928

[6] Kim J-R Kim HA. Molecular mechanisms of sex-related differences in arthritis and associated pain. Int J Mol Sci. (2020) 21:7938. doi: 10.3390/ijms21217938 33114670 PMC7663489

[7] Novais M Henriques T Vidal-Alves MJ Magalhães T. When problems only get bigger: the impact of adverse childhood experience on adult health. Front Psychol. (2021) 12:693420. doi: 10.3389/fpsyg.2021.693420 34335410 PMC8318698

[8] O'Mahony J Bernstein CN Marrie RA. Adverse childhood experiences and psychiatric comorbidity in multiple sclerosis, inflammatory bowel disease, and rheumatoid arthritis in the Canadian longitudinal study on aging. J Psychosom Res. (2024) 187:111893. doi: 10.1016/j.jpsychores.2024.111893 39306899

[9] Luiz APL Antico HA Skare TL Boldt ABW Nisihara R. Adverse childhood experience and rheumatic diseases. Clin Rheumatol. (2018) 37:2863–7. doi: 10.1007/s10067-018-4200-5 29992393

[10] Li L Yang M Manson S O'Connell J Jiang L. Associations of hearing loss with subjective cognitive decline among American Indians and Alaska Natives and other race/ethnic groups: results from the BRFSS. Alzheimer Dement. (2024) 20:e093052. doi: 10.1002/alz.093052

[11] Testa A Semenza D Jackson DB Fu K McKay S Ganson KT . Social isolation and firearm secure storage in the USA: results from the 2022 BRFSS. Inj Prev. (2024) ip−2024–045468. doi: 10.1136/ip-2024-045468 39578054

[12] Rubinstein TB Bullock DR Ardalan K Mowrey WB Brown NM Bauman LJ . Adverse childhood experiences are associated with childhood-onset arthritis in a national sample of US youth: an analysis of the 2016 national survey of children's health. J Pediatr. (2020) 226:243–50.e2. doi: 10.1016/j.jpeds.2020.06.046 32553837

[13] Lin L Wang HH Lu C Chen W Guo VY. Adverse childhood experiences and subsequent chronic diseases among middle-aged or older adults in China and associations with demographic and socioeconomic characteristics. JAMA Netw Open. (2021) 4:e2130143. doi: 10.1001/jamanetworkopen.2021.30143 34694390 PMC8546496

[14] Felitti VJ Anda RF Nordenberg D Williamson DF Spitz AM Edwards V . Relationship of childhood abuse and household dysfunction to many of the leading causes of death in adults. Am J Prev Med. (1998) 14:245–58. doi: 10.1016/S0749-3797(98)00017-8 9635069

[15] Piercy KL Troiano RP Ballard RM Carlson SA Fulton JE Galuska DA . The physical activity guidelines for Americans. JAMA. (2018) 320:2020. doi: 10.1001/jama.2018.14854 30418471 PMC9582631

[16] Huang J Spira AP Perrin NA Ellis A Hsu EC Kaufmann CN . Latent classes of sleep problems and subjective cognitive decline among middle-aged and older adults in the United States. Arch Gerontol Geriatr. (2025) 129:105657. doi: 10.1016/j.archger.2024.105657 39405666

[17] Jia H Lubetkin EI. Ranking the ten adverse childhood experiences: long-term consequences to health-related quality of life. Am J Prev Med. (2024) 67:265–73. doi: 10.1016/j.amepre.2024.04.001 38599501

[18] Badley EM Shields M O'Donnell S Hovdestad WE Tonmyr L. Childhood maltreatment as a risk factor for arthritis: findings from a population-based survey of Canadian adults. Arthrit Care Res. (2019) 71:1366–71. doi: 10.1002/acr.23776 30328298

[19] Konjikusic A Ohrndorf S Braun T Köhler V Höhne-Zimmer V Schmittat G . AB0295 higher prevalence of depression with link to childhood trauma in an early arthritis cohort – a selective data analysis (PSYRA-study). Ann Rheum Dis. (2022) 81:1273. doi: 10.1136/annrheumdis-2022-eular.341535609976

[20] Li S Chen W Sun D Fernandez C Li J Kelly T . Variability and rapid increase in body mass index during childhood are associated with adult obesity. Int J Epidemiol. (2015) 44:1943–50. doi: 10.1093/ije/dyv202 26452389 PMC4715253

[21] Felitti VJ Anda RF Nordenberg D Williamson DF Spitz AM Edwards V . Relationship of childhood abuse and household dysfunction to many of the leading causes of death in adults. Am J Prev Med. (1998) 14:245–58. doi: 10.1016/S0749-3797(98)00017-8 9635069

[22] Seror R Gusto G Boutron-Ruault M Mariette X. OP0253 Passive smoking in childhood and history of chronic diarrhea increases the risk of developing rheumatoid arthritis (RA). Ann Rheum Dis. (2017) 76:160–1. doi: 10.1136/annrheumdis-2017-eular.3853 30124939

[23] Dube SR Fairweather D Pearson WS Felitti VJ Anda RF Croft JB. Cumulative childhood stress and autoimmune diseases in adults. Psychosom Med. (2009) 71:243–50. doi: 10.1097/PSY.0b013e3181907888 19188532 PMC3318917

[24] Miller GE Chen E Parker KJ. Psychological stress in childhood and susceptibility to the chronic diseases of aging: moving toward a model of behavioral and biological mechanisms. Psychol Bull. (2011) 137:959–97. doi: 10.1037/a0024768 21787044 PMC3202072

[25] Anda RF Felitti VJ Bremner JD Walker JD Whitfield C Perry BD . The enduring effects of abuse and related adverse experiences in childhood: a convergence of evidence from neurobiology and epidemiology. Eur Arch Psychiatry Clin Neurosci. (2006) 256:174–86. doi: 10.1007/s00406-005-0624-4 16311898 PMC3232061

[26] Kavelaars A Heijnen CJ. T cells as guardians of pain resolution. Trends Mol Med. (2021) 27:302–13. doi: 10.1016/j.molmed.2020.12.007 33431239 PMC8005447

[27] Heluany CS De Palma A Day NJ Farsky SHP Nalesso G. Hydroquinone, an environmental pollutant, affects cartilage homeostasis through the activation of the aryl hydrocarbon receptor pathway. Cells. (2023) 12:690. doi: 10.3390/cells12050690 36899825 PMC10001213

[28] Watari T Naito K Sakamoto K Kurosawa H Nagaoka I Kaneko K. Evaluation of the effect of oxidative stress on articular cartilage in spontaneously osteoarthritic STR/OrtCrlj mice by measuring the biomarkers for oxidative stress and type II collagen degradation/synthesis. Exp Ther Med. (2011) 2:245–50. doi: 10.3892/etm.2011.196 22977492 PMC3440670

[29] Fernández-Torres J Aztatzi-Aguilar OG Zamudio-Cuevas Y Sierra-Vargas MP Martínez-Nava GA Montaño-Armendáriz N . Effect of smoking on the redox status of knee osteoarthritis: a preliminary study. Exp Biol Med. (2023) 248:1754–67. doi: 10.1177/15353702231199072 37916410 PMC10792422

[30] Yudoh K Nguyen vT Nakamura H Hongo-Masuko K Kato T Nishioka K. Potential involvement of oxidative stress in cartilage senescence and development of osteoarthritis: oxidative stress induces chondrocyte telomere instability and downregulation of chondrocyte function. Arthritis Res Ther. (2005) 7:R380. doi: 10.1186/ar1499 15743486 PMC1065334

[31] Schiller J Fuchs B Arnhold J Arnold K. Contribution of reactive oxygen species to cartilage degradation in rheumatic diseases: molecular pathways, diagnosis and potential therapeutic strategies. Curr Med Chem. (2003) 10:2123–45. doi: 10.2174/0929867033456828 12871089

[32] Vallerand IA Lewinson RT Frolkis AD Lowerison MW Kaplan GG Swain MG . Depression as a risk factor for the development of rheumatoid arthritis: a population-based cohort study. RMD Open. (2018) 4:e000670. doi: 10.1136/rmdopen-2018-000670 30018804 PMC6045711

[33] Kobayashi MA Isasi CR Suglia SF Gallo LC Gutierrez AP Sotres-Alvarez D . Adverse childhood experiences and adult disease: examining mediating pathways in the hispanic community health study/study of Latinos sociocultural ancillary study. Health Psychol. (2024) 43:627–38. doi: 10.1037/hea0001349 38884976 PMC12004413

[34] Deschênes SS Kivimaki M Schmitz N. Adverse childhood experiences and the risk of coronary heart disease in adulthood: examining potential psychological, biological, and behavioral mediators in the Whitehall II cohort study. J Am Heart Assoc. (2021) 10:e019013. doi: 10.1161/JAHA.120.019013 33938232 PMC8200717

